# Rapid detection of antimicrobial resistance in methicillin-resistant *Staphylococcus aureus* using MALDI-TOF mass spectrometry

**DOI:** 10.3389/fcimb.2023.1281155

**Published:** 2023-11-23

**Authors:** Josiah J. Rensner, Paul Lueth, Bryan H. Bellaire, Orhan Sahin, Young Jin Lee

**Affiliations:** ^1^ Department of Chemistry, Iowa State University, Ames, IA, United States; ^2^ Department of Veterinary Microbiology and Preventative Medicine, Iowa State University, Ames, IA, United States; ^3^ Department of Veterinary Diagnostic and Production Animal Medicine, Iowa State University, Ames, IA, United States

**Keywords:** MRSA, deuterium, antimicrobial resistance, MALDI, mass spectrometry, Biotyper

## Abstract

Antimicrobial resistance is a growing problem in modern healthcare. Most antimicrobial susceptibility tests (AST) require long culture times which delay diagnosis and effective treatment. Our group has previously reported a proof-of-concept demonstration of a rapid AST in *Escherichia coli* using deuterium labeling and MALDI mass spectrometry. Culturing bacteria in D_2_O containing media incorporates deuterium in newly synthesized lipids, resulting in a mass shift that can be easily detected by mass spectrometry. The extent of new growth is measured by the average mass of synthesized lipids that can be correlated with resistance in the presence of antimicrobials. In this work, we adapt this procedure to methicillin-resistant *Staphylococcus aureus* using the Bruker MALDI-TOF Biotyper, a low-cost instrument commonly available in diagnostic laboratories. The susceptible strain showed a significant decrease in average mass in on-target microdroplet cultures after 3 hours of incubation with 10 µg/mL methicillin, while the resistant strain showed consistent labeling regardless of methicillin concentration. This assay allows us to confidently detect methicillin resistance in *S. aureus* after only 3 hours of culture time and minimal sample processing, reducing the turn-around-time significantly over conventional assays. The success of this work suggests its potential as a rapid AST widely applicable in many clinical microbiology labs with minimal additional costs.

## 1 Introduction

Antimicrobial resistance (AMR) is a growing problem for modern healthcare. AMR infections are one of the leading causes of death worldwide, with an estimated 1.27 million deaths attributed to AMR infections in 2019 (Murray et al., 2022). Many of these cases are contracted in hospitals, and increased hospitalization during the COVID-19 pandemic has exacerbated this issue (U.S. Center for Disease Control, 2022). This is especially notable in a group of bacteria collectively referred to as the ESKAPE pathogens (*Enterococcus faecium*, *Staphylococcus aureus*, *Klebsiella pneumoniae*, *Acinetobacter baumannii*, *Pseudomonas aeruginosa*, and *Enterobacter* spp.) that are leading causes of hospital infections and are frequently drug-resistant (Mulani et al., 2019). Progress to develop new antibiotics has been slow (Terreni et al., 2021), suggesting the need for innovations in AMR research.

Successful treatment of resistant infections requires both species identification of the causative bacteria and antimicrobial susceptibility testing (AST) (Leonard et al., 2018). Species identification has long been established, notably with polymerase chain reaction (Barghouthi, 2011; Kralik and Ricchi, 2017) and more recently with mass spectrometry methods (Claydon et al., 1996; Li et al., 2022), largely replacing laborious biochemical assays. The Bruker Biotyper (Zhu et al., 2015) and BioMérieux Vitek MS (Girard et al., 2021) utilize bench-top matrix-assisted laser desorption/ionization time-of-flight (MALDI-TOF) mass spectrometers and have been approved by the U.S. Food and Drug Administration (FDA) for automated bacterial species identification. AST is more difficult and requires more innovation. The traditional methods for determining antimicrobial resistance are broth and agar dilutions (Wiegand et al., 2008), which involve culturing bacteria with different concentrations of antibiotics and using colony counting or optical spectroscopy to determine growth. This is reliable, but it requires long culture times, typically overnight, which results in a long assay turnaround time (TAT). PCR-based methods have also been developed (Sundsfjord et al., 2004), but they generally require targeting known resistance genes, and so are not useful in all instances. A recent development combines short culturing in the presence of antibiotics with qPCR and melt analysis for in-parallel bacterial identification and AST (Athamanolap et al., 2017). Other methods have recently gotten FDA approval for AST, including the REVEAL^®^ Rapid Antimicrobial Susceptibility Test System (Tibbetts et al., 2022) and the Selux NGP System (U.S. Food and Drug Administration, 2023).

Mass spectrometry (MS) measures the mass-to-charge ratios of ionized molecules and is extensively used to study biological samples (Lössl et al., 2016; Aksenov et al., 2017). Bacterial species identification by benchtop MALDI-TOF MS is performed by matching protein signals with protein fingerprint patterns (Li et al., 2022). This has become a popular assay commonly used in clinical diagnostic laboratories; however, it does not provide any resistance information. Bruker have developed the MBT-STAR assay for the Biotyper instrument that can measure β-lactam resistance by detecting the hydrolysis products of β-lactam drugs (Kawamoto et al., 2018). While this is useful, it only applies to this specific drug class and resistance mechanism; therefore, it is not universally applicable. Other ASTs have been developed using mass spectrometry (Florio et al., 2020), but they generally involve tracking specific biomarkers, which is only applicable to a specific species and resistance mechanism. Other efforts have also been made to use the Biotyper for AST in parallel with the standard identification assay. Notably, one approach uses the Biotyper by growing bacteria in microdroplet cultures directly on a MALDI target plate and correlating the detection of sufficient amount of proteins with bacterial growth (Idelevich et al., 2018a). This method was successfully applied to *Enterobacterales* (Idelevich et al., 2018b) and *S. aureus* in positive blood cultures (Nix et al., 2020); however, it is subject to false positives as it relies on a raw signal threshold to determine resistance.

Mass spectrometric analysis with stable isotope labeling has shown promise for AST since it creates a mass shift in molecules synthesized by newly grown cells (Demirev et al., 2013; Jung et al., 2014). These previous approaches rely on protein profiling using ^13^C (and ^15^N), which limits their ease of application since proteins vary significantly depending on the species. These approaches also cannot measure minimum inhibitory concentration (MIC) of an antibiotic. We have previously reported an MS-based, rapid AST using deuterium labeling of bacterial lipids in on-target microdroplet cultures and demonstrated it for *Escherichia coli* treated with ciprofloxacin (Larson et al., 2022). When bacteria are cultured in deuterium oxide (D_2_O)-containing media, the heavy deuterium isotope is incorporated into newly synthesized membrane lipids which distinguishes them from unlabeled pre-existing lipids. We measure this incorporation by calculating an average mass of all isotopologues, including natural isotopes and added deuterium. By monitoring deuterium incorporation from the average of individual lipids, we could not only distinguish susceptible vs. resistant *E. coli*, but also measure the MIC for each strain. In this work, we expand our previous proof-of-concept study to a clinically useful tool. Firstly, we demonstrate this method for methicillin-resistant *S. aureus*, one of the ESKAPE pathogens that has clinical relevance (Kourtis, 2019). As a Gram-positive species, this is also an extension of our previous work using Gram-negative *E. coli*. Most importantly, we adapt this protocol to Bruker’s newest MALDI Biotyper with negative ion mode capability (MBT sirius), greatly increasing its accessibility in clinical settings.

## 2 Methods

### 2.1 Sample preparation

To conduct these experiments, methicillin-susceptible *Staphylococcus aureus* Seattle 1945 (catalog number 25923) and methicillin-resistant *Staphylococcus aureus* 328 (catalog number 33591) were purchased from American Type Culture Collection (Manassas, VA). The bacteria were cultured overnight in a 37°C incubator on Columbia blood agar plates (Becton, Dickson and Company, Sparks, MD). A sample removed from the agar was suspended in Mueller Hinton broth (Becton, Dickson and Company) and diluted to a concentration of 8x10^8^ cfu/mL using an OD_600_ measurement and a correction factor of 0.1 OD = 5x10^8^ cfu/mL. Samples were prepared on MBT Biotarget 96 MALDI target plates (Bruker, Billerica, MA) and SGT µFocus plates (Hudson Surface Technology, Closter, NJ) for MBT MALDI-TOF and MALDI-Orbitrap, respectively. High-purity deuterium oxide (D atom 99.9%; Cambridge Isotope Laboratories, Tewksbury, MA) was diluted to 40% D_2_O using deionized water. A 2.5 µL volume of broth culture and 40% D_2_O with varying methicillin concentrations were spotted onto the MBT target plates, while 3 µL volume of the broth culture and 3 µL of the D_2_O solutions were spotted onto µFocus plates. This results in final cultures suspended in 0.5x Mueller Hinton broth with 20% D_2_O and a 4x10^8^ cfu/mL initial bacterial concentration, with a total volume of 5 µL for MBT samples and 6 µL for µFocus samples. Samples were placed in incubation chambers modified from Bruker MALDI target sample holders with 4 mL of 20% D_2_O added to the base and were sealed by wrapping with Parafilm M (Amcor, Victoria, Australia). Samples were cultured in a 37°C incubator for 2-4 hours.

**Figure 1:**
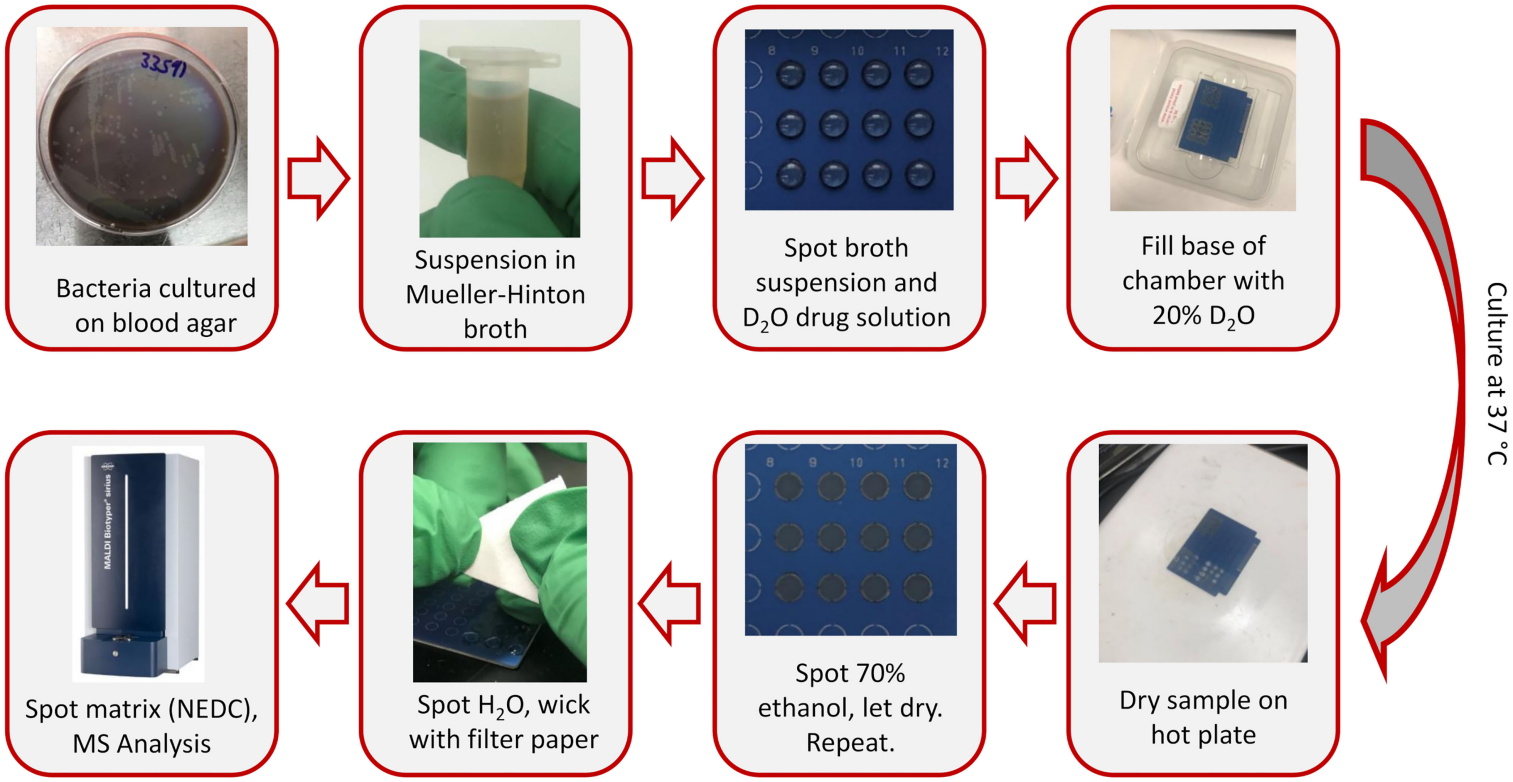
AST workflow for bench-top MALDI-TOF analysis of on-target deuterium-labeling.

After incubation samples were dried using a hot plate (~75°C). The samples were removed from heat and then two treatments of 70% ethanol were spotted onto the samples to lyse the cells, allowing the samples to completely dry between applications. After the ethanol had dried, water was added and then after 30 seconds was wicked off using filter paper (Whatman Grade 1, Cytiva, Marlborough, MA). A 1 µL volume of 70% ethanol and 3 µL of water was used for the MBT plates and 3 µL volumes were used for both treatments for the µFocus plates. Our previous protocol used 100% ethanol as the lysing agent and 1% trifluoroacetic acid (TFA) for wicking. Using 70% ethanol in place of neat ethanol prevents the solvent from spreading across the MBT plates and potential cross-contamination with other cultures spots. Additionally, avoiding the use of TFA minimized the ion suppression of phosphatidylglycerol (PG) ion signal in negative ion mode and using water instead removed contaminants without the side effect. N-(1-naphthyl) ethylenediamine dihydrochloride (Tokyo Chemical Industry US, Portland, OR) was used as the MALDI matrix. A 10 mg/mL matrix solution was prepared in 50% methanol and 2 and 3 µL was spotted onto the MBT plate and µFocus plates samples, respectively. Samples were placed on a hot plate (~75°C) during matrix application to ensure fast solvent evaporation and even crystal formation. This workflow is represented in **Figure 1**.

### 2.2 Mass spectrometry data collection and analysis

Samples on the MBT target plate were analyzed using flexControl software (version 3.4; Build 207.20) in negative ion mode using a Biotyper sirius MALDI-TOF mass spectrometer (Bruker, Billerica, MA). The LN_PepMix method for analyzing small molecules was used with the following voltage parameters: Ion Source 1 = -10 kV, Ion Source 2 = -9 kV, Lens = -3 kV. The LN_PepMix method was modified by increasing the laser power and changing the analyzed mass range. Laser power was set at 80% but varied from spot to spot to get an optimal signal to noise ratio for each spectrum. The mass range was adjusted to *m/z* 680-820 to detect phospholipids.

Resulting spectra were opened using MSConvertGUI (Chambers et al., 2012) and converted to mzXML files. The mzXML files were then opened using mMass Version 5.5.0 (Niedermeyer and Strohalm, 2012). Batch processing peak picking was done with a signal/noise threshold of 1, an intensity threshold of 0, and a picking height of 100. This ensured that all peaks were automatically selected and their *m/z* and intensity values could be copied into Excel. Data was post-calibrated using PG 34:1 standard spotted separately and analyzed in parallel. All samples were prepared in triplicate. This raw data is included in the **Supplementary Information**.

For high-mass resolution Orbitrap analysis, samples on the µFocus plate were analyzed in negative ion mode using an Orbitrap Q Exactive HF mass spectrometer (Thermo Scientific, San Jose, CA) with a Spectroglyph MALDI source (Spectroglyph, Kennewick, WA) equipped with a 349 nm laser (Explorer One; Spectra Physics, Milpitas, CA) using a 500 Hz repetition rate and ~4 µJ per shot. Samples were analyzed at 120,000 resolving power at *m/z* 200 for a 700-800 *m/z* range. A 60 µm raster step was used for the entire sample spot (11 minute acquisition) to ensure a good representation of the sample and the resulting spectra were averaged in Xcalibur. Peak lists from averaged spectra were exported to .csv files and used to calculate average mass of the lipids of interest. All samples were prepared in triplicate.

## 3 Results

In the previous demonstration of D-labeling AST, we targeted phosphatidylethanolamines (PEs) in positive ion mode (Larson et al., 2022) as they are the major membrane lipids of *E. coli* (Sohlenkamp and Geiger, 2016). Since phosphatidylglycerols (PGs) comprise the majority of membrane lipids in *S. aureus* (Young et al., 2019), we adapted our previous protocol for negative ion mode analysis since PGs are more readily detected as deprotonated negatively charged species. Additionally, we adapt this protocol for the Biotyper MALDI-TOF, with the adjusted workflow summarized in **Figure 1**. NEDC, a recently developed matrix effective in negative ion mode (Chen et al., 2012), was used as a MALDI matrix instead of previously used 2,5-dihydroxybenzoic acid. Additionally, the solvent used for wicking away broth background ions was changed from 1% TFA to pure water, since residual acid suppressed deprotonated ion signal. The use of negative ion mode and the analysis of lipids is a departure from the conventional Biotyper assay, analyzing proteins in positive ion mode. We modified an analysis method provided by the Bruker for small molecule analysis by increasing the laser power and altering the mass range.

**Figure 2:**
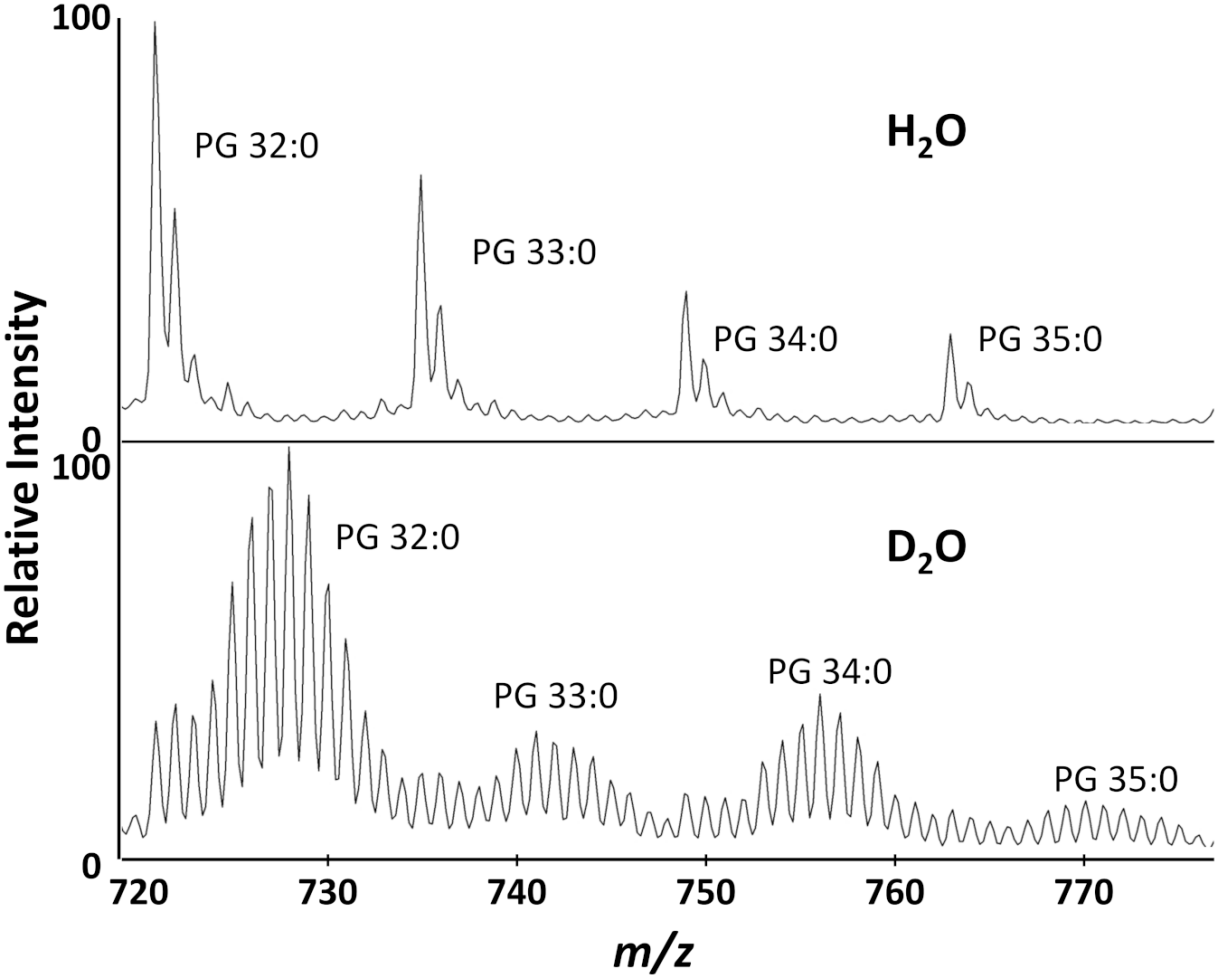
Biotyper mass spectra for the major lipids of *S. aureus* grown in H_2_O or 20% D_2_O microdroplet cultures for 4 hours.

We were able to detect multiple PGs (**Figure 2**), but chose to use PG 32:0, the most abundant lipid, as the primary target for data analysis. After *S. aureus* is grown in D_2_O, the monoisotopic peaks of membrane lipids are much smaller and the mass spectrum is dominated by a binomial distribution of deuterium labeled peaks. For PG 32:0, this distribution averages to about seven incorporated deuterium atoms, corresponding to ~50% deuterium labeling efficiency out of 72 carbon-bound hydrogens with 20% D_2_O. A similar D-labeling efficiency was previously observed in PEs in *E. coli* (Larson et al., 2022), typical of the kinetic isotope effect in *in vivo* deuterium labeling of bacteria. Namely, newly synthesized lipids have ~10% (~50% D-labeling efficiency x 20% D_2_O concentration) of C-H replaced by C-D, clearly separated from the “old lipids” in the initial cells that started the culture. Some of the unlabeled lipids remain, but a majority of PG 32:0 is from the new lipids that incorporate deuterium.

While application to the Bruker Biotyper was the main focus of this study, data was also collected on a high-resolution Orbitrap MS, which was used in our previous work, for comparison (**Figure S1**). High resolution data allows determination of the molecular formulae of our analytes, resulting in confident metabolite annotation. This also allows us to easily separate labeled lipids from other interfering molecules present in the samples. The overall results between the two instruments are comparable, verifying that a low-resolution time of flight instrument is sufficient for this analysis. There are some contamination peaks that are detected only in the Orbitrap, but their presence seems minimal in the Biotyper spectrum and would not affect the results.

**Figure 3:**
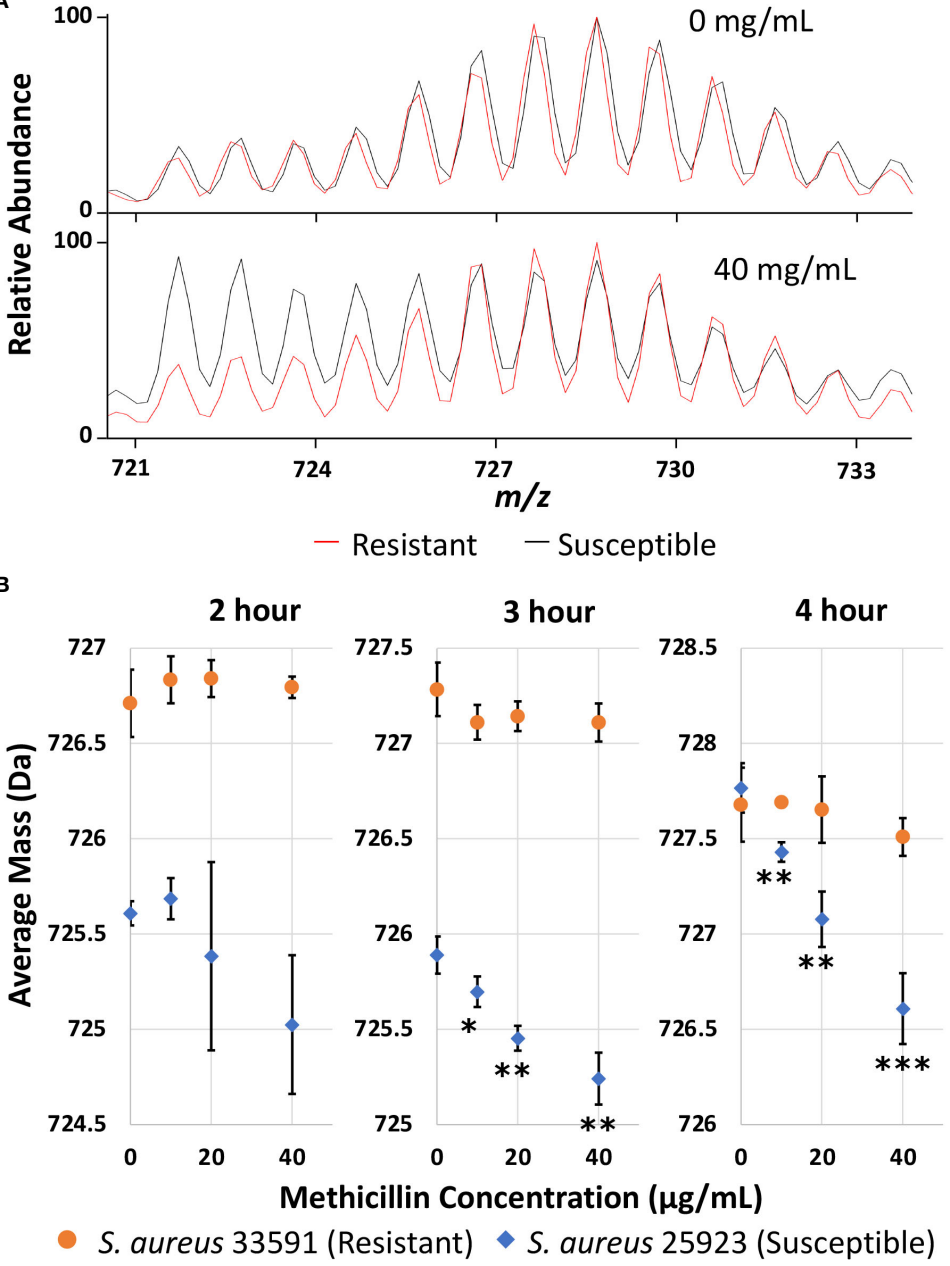
**(A)** Representative mass spectra of PG 32:0 from the resistant and susceptible *S. aureus* grown for 4 hours in 20% D_2_O microdroplet cultures with and without 40 mg/mL methicillin. **(B)** Average mass of PG 32:0 in *S. aureus* measured by the Bruker Biotyper from 20% D_2_O microdroplet cultures with varying methicillin concentrations and culture times. The asterisks indicate significance with *, **, and *** corresponding to p-values of <0.05, <0.01, and <0.001 respectively when compared to the untreated control.

Once experimental conditions were optimized to monitor the major lipid species in *S. aureus* and efficient D-labeling was confirmed, further experiments were performed to demonstrate AMR detection. Methicillin resistant and susceptible *S. aureus* strains were cultured as microdroplets on MALDI target plates at 37°C in 0.5x Mueller Hinton broth with 20% D_2_O for 2, 3, and 4 hours with 0-40 µg/mL methicillin. Representative Biotyper mass spectra are shown in **Figures S2–S4** for each time point and methicillin concentration. Unlike *E. coli*, *S. aureus* grows slowly and there are a significant amounts of unlabeled “old lipids” in 2 or 3 hours of culture even without any antibiotics. To illustrate how our AST works, **Figure 3A** compares the representative mass spectra from 4-hour culture comparing treated and untreated samples. Without antibiotics, both susceptible and resistant strains show similar spectra dominated by newly grown D-labeled lipids. While MRSA shows almost no difference when treated with methicillin, the susceptible strain has a minimal new growth with unlabeled “old lipids” comparable to that of D-labeled.

The relative abundance of D-labeling can be estimated from the change of average mass for each specific lipid. Average mass, defined as ( ∑ M_i_ I_i_)/( ∑ I_i_), is a good measure of how much new synthesis occurred compared to the original loading. Here, M_i_ and I_i_ represent the mass and intensity of peaks with i number of D-labeling, respectively, but i can also include ^13^C or other natural isotopes due to insufficient mass resolution. In the absence of new growth in deuterated media, the average mass would be the same as that in an unlabeled control, while new lipid synthesis in D_2_O containing media increases the average mass. The average mass is used as an easily quantifiable measure in this study to represent new growth in the presence of antibiotics. As shown in **Figure 3B**, the average mass for the resistant strain does not change regardless of methicillin concentration, while it decreases monotonically for the susceptible strain. The decrease is insignificant with a 2-hour culture time due to the large experimental error, but is clearly observed after 3 or 4 hours. The treated cultures were compared to the untreated culture for each strain with their p-values summarized in **Table S1**. For untreated samples, longer cultures times increase the portion of new lipids for both strains, as indicated by the higher average mass. The susceptible strain seems to have a longer lag time than the resistant strain when introduced to the broth media, as seen by the lower average mass after two or three hours of culturing that becomes similar after 4 hours. Crucially, the measured average mass clearly decreases as methicillin concentration increases after 3 or 4 hours for the susceptible strain, suggesting differentiation of susceptible and resistant *S. aureus* is possible after only 3 hours of culturing. The same result was obtained on the Orbitrap MS as shown in **Figure S5**. Other membrane lipids show similar results, as shown for PG 33:0 and PG 34:0 in **Figure S6**, albeit with more error due to lower signal.

## 4 Discussion

Stable isotopes have long been used for biological discovery and biomedical research. Their usage in life sciences has accelerated with the advent of biological mass spectrometry (Lehmann, 2017). Tracing *in vivo* labeled stable isotopes with mass spectrometry has become a powerful tool in metabolic flux analysis and the study of metabolic pathways (Freund and Hegeman, 2017; De Feyter and de Graaf, 2021). Deuterium is a versatile tracer that can be incorporated into most biomolecules by simply replacing H_2_O with D_2_O in the growing medium (Kim et al., 2023). Nicotinamide adenine dinucleotide phosphate (NADPH) is the primary cellular reductant in most biosynthesis and half of NADPH’s redox active hydrogen is the result of enzyme-catalyzed hydrogen transfer from water (Zhang et al., 2017). D-labeling is especially well suited for tracing fatty acids and lipids, as fatty acyl chains are reduced by ACP reductases (Kazuki et al., 1980). Single-cell Raman spectroscopy (Song et al., 2017; Zhang et al., 2020) and Fourier-Transform Infrared Spectroscopy (Shams et al., 2023) were able to detect AMR by monitoring C-D vibrations but these approaches require specialized equipment. This work successfully demonstrated a simple adaptation of a commonly available bench-top MALDI-TOF for rapid AMR detection of MRSA using D-labeling.

D_2_O is toxic, but most bacteria grow well in up to 50% D_2_O with no apparent adverse effects (Kushner et al., 1999). In our AMR assay, the D_2_O concentration can be varied to change the mass shift of labeled peaks and minimize overlap with other ions. With ~50% D-labeling efficiency, 20% D_2_O concentration was the most effective, resulting in an average peak shift of ~7 Da for D-labeled peaks where there is minimal interference (**Figure 2**). There are some ^13^C_1_ and ^13^C_2_ natural isotope peaks present in the unlabeled lipids that cannot be distinguished from new deuterium labeled lipids with a low mass resolution MALDI-TOF. However, it is unnecessary to separate them for our purpose as they will all be simply summed together when calculating average mass. It is important to confirm there is little interference in the analyzed mass range of unlabeled control samples to ensure that the average mass would not be significantly affected by contaminants in the labeled samples.

Average mass is a good measure of the relative abundance of old vs new lipids. Namely, average mass can be re-expressed as (1-*f*) MW_0_ + *f* MW_D_, where MW_0_, MW_D_, and *f* represent the molecular weight (MW) of unlabeled old lipids, MW of labeled new lipids, and the fraction of new lipids (= ∑ _new_ I_i_/ ∑ _all_ I_i_). The MW in the mass spectrum is one Dalton less than the neutral mass due to deprotonation. As MW_0_ is 722.1 Da from the unlabeled spectrum and MW_D_ is 729.3 Da assuming 50% D-labeling efficiency, *f* is 0.65, 0.69, and 0.76 for the resistant *S. aureus* with 2-, 3-, and 4-hour cultures, indicating that a significant fraction is already D-labeled after 2 hours. Therefore, there is a potential to minimize the culture time to 2 hours if we can minimize experimental error. It should be noted that a significant fraction is also D-labeled even for the susceptible strain treated with 40 µg/mL methicillin, *f* of 0.41, 0.44, and 0.61, respectively, for 2-, 3-, and 4-hour cultures, due to bacterial growth before they are killed by the antibiotic. Nevertheless, p-values of 0.053, 0.0018, and 0.00074 were obtained for 2-, 3- and 4-hour cultures, respectively, when comparing the average mass of the susceptible strain for untreated samples and samples treated with 40 µg/mL methicillin, again suggesting AST is possible with a 3-hour culture but may be reduced to a 2 hour culture if the error can be minimized.

Microdroplet culture spots on MALDI target plates allow for parallel culturing of multiple drug concentrations and rapid MS analysis. This sample preparation was developed by the Becker group for a protein-based AMR assay (Idelevich et al., 2018a; Nix et al., 2020), but we adapted it for an AMR assay based on deuterium labeling of bacterial lipids. Their AMR assay uses the standard Biotyper assay, which acquires protein mass spectra in positive ion mode and searches a bacterial database. They start with a low concentration of bacteria in the presence of antibiotics and a matching score greater than 1.7 is classified as resistant. As it relies on an arbitrary score as an absolute cutoff point, there are several potential pitfalls in this approach. Firstly, the choice of starting concentration (e.g., 5x10^5^ cfu/mL used by the Becker group) is important, which should be low enough not to be detected by the Biotyper assay initially, but high enough to grow to a consistently detectable population after a few hours of culturing. Unfortunately, growth rates can vary significantly depending on bacterial species (Gibson et al., 2018), resulting in false positive or false negative readings. Secondly, this type I or type II error is exacerbated by Boolean decisions, instead of providing a probability or p-value. Lastly, it cannot be applicable to bacterial species that are not present in the database. In contrast, our approach relies on the relative value, or ratio, between unlabeled and labeled membrane lipids. We arbitrarily chose a high starting concentration in this work, 8x10^8^ cfu/mL, to have sufficient lipid signals even when there is no growth, but this concentration can be varied as long as there are sufficient signals. Furthermore, as we discussed above, we can provide p-value for a strains’ resistance by comparing the average masses of a control sample vs a sample treated with a high antibiotic concentration.

When combined with our previous work using *E. coli* (Larson et al., 2022), its successful application to *S. aureus* indicates that this method could be adapted to both Gram-positive and Gram-negative strains. Although it is yet to be proven, if the major membrane lipids are relatively similar between bacterial species, for example mostly PGs or PEs, our approach could be adapted to almost any bacteria with only minor changes in data processing. Simultaneous species identification is an advantage of the Becker group’s AMR assay, but it takes only a few more minutes for our assay to perform species identification from an additional culture spot as we are using the same instrument and sample plate. One limitation of the current work is that it was tested only for a single susceptible and resistant strains as a proof-of-concept study. However, it will be tested against multiple MRSA strains in the future to provide statistical data across a wider strain collection.

## 5 Conclusions

In conclusion, we have taken our previously demonstrated deuterium labeling bacterial antimicrobial resistance assay and adapted it to the Bruker MALDI Biotyper widely available in clinical microbiology labs. Additionally, we have shown that our approach is also applicable to a Gram-positive bacterium using negative ion mode, specifically methicillin-resistant *S. aureus*, a relatively slow-growing ESKAPE pathogen. While the targeted lipids could be slightly different for each bacterial species, this approach should be universally applicable with only minor procedure adjustments. There is much additional work to be done, including application to other ESKAPE pathogens, evaluation in a large cohort of clinical isolates, and integration into a routine AST workflow. However, we envision that this assay can be eventually generalized to any AMR pathogens, regardless of Gram-designation, including known and unknown bacterial species, while simultaneously performing bacterial identification from an additional spot on the same sample plate by protein profiling. This both increases the accessibility of our developed assay and could also increase diagnostic labs’ capability with no additional instrumentation and minimal additional materials.

## Data availability statement

The raw data supporting the conclusions of this article are available as a supplementary zip file.

## Author contributions

JR: Data curation, Investigation, Methodology, Writing – original draft, Writing – review & editing. PL: Investigation, Writing – review & editing. BB: Resources, Supervision, Writing – review & editing. OS: Funding acquisition, Resources, Supervision, Writing – review & editing. YL: Conceptualization, Funding acquisition, Methodology, Project administration, Supervision, Writing – original draft, Writing – review & editing.
