# Supplementary material for: Rapid detection of antimicrobial resistance in methicillin-resistant Staphylococcus aureus using MALDI-TOF mass spectrometry
Source: Front Cell Infect Microbiol. 2023 Nov 23;13:1281155. doi: 10.3389/fcimb.2023.1281155 (PMC10702551; doi:10.3389/fcimb.2023.1281155)
Supplement: Supplementary file 1 [file DataSheet_1.pdf]

Supplementary Material for

Rapid Detection of Antimicrobial Resistance in methicillin-resistant *Staphylococcus aureus* Using Deuterium Labeling and the Biotyper MALDI-TOF MS

Supplementary Figures

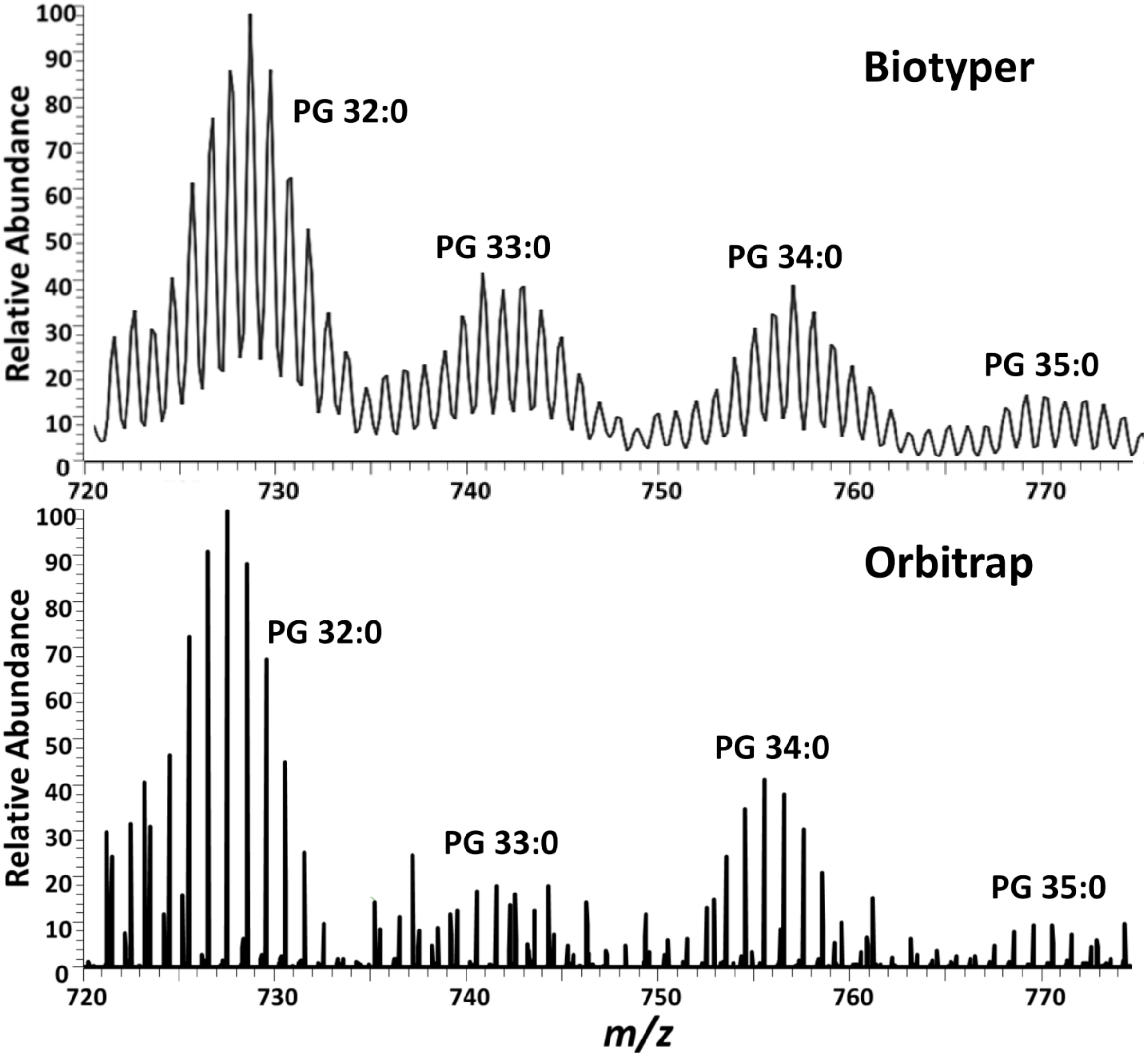

Figure S1. Comparison of Biotyper and Orbitrap mass spectra for the major lipids of untreated methicillin-susceptible *S. aureus* Seattle 1945 after 4 hours of microdroplet culture in 20% D_2O.

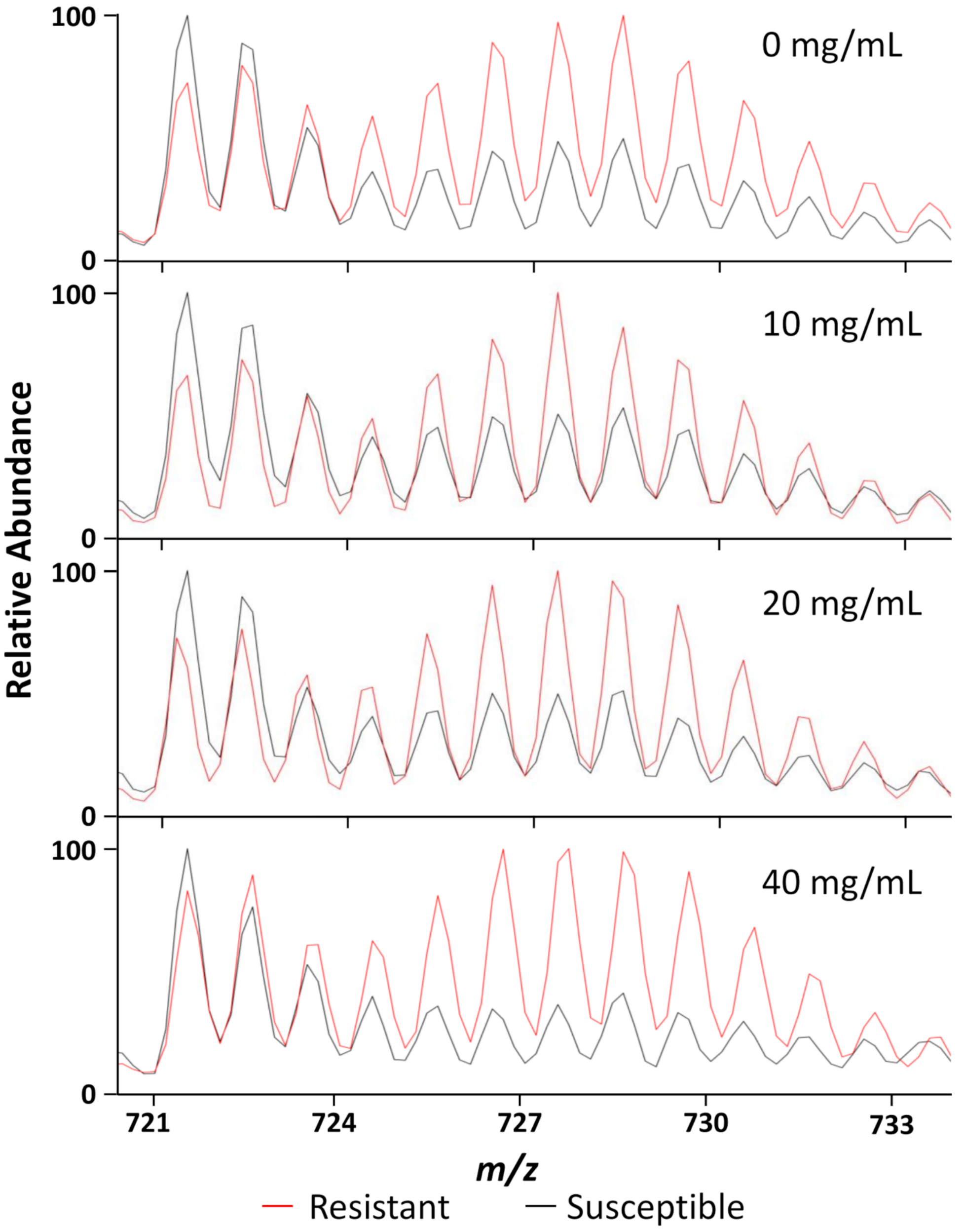

Figure S2. Representative Biotyper mass spectra of methicillin-resistant and methicillin-susceptible strains grown in 20% D_2O for 2 hours with varied methicillin concentrations.

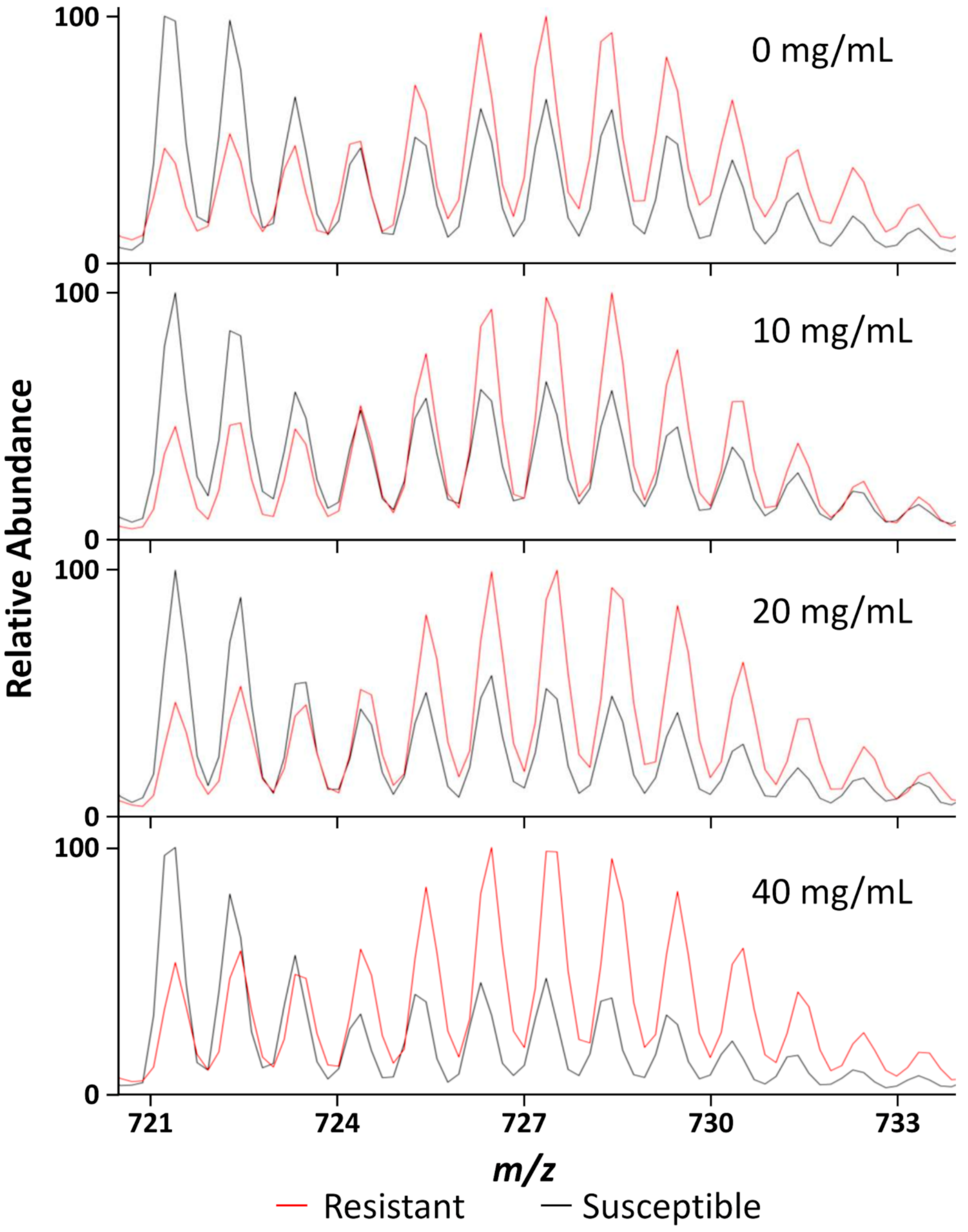

Figure S3. Representative Biotyper mass spectra of methicillin-resistant and methicillin-susceptible strains grown in 20% D_2O for 3 hours with varied methicillin concentrations.

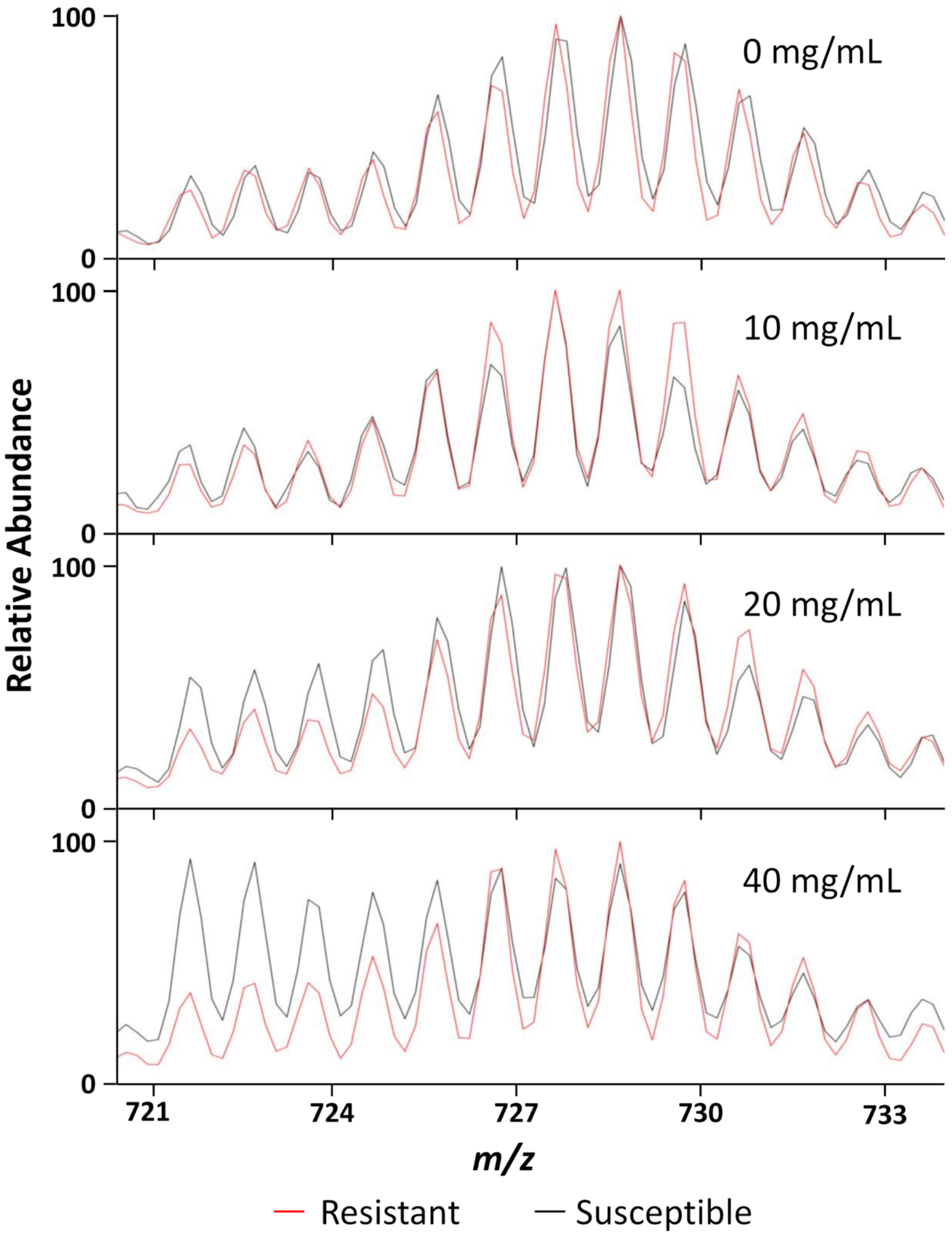

Figure S4. Representative Biotyper mass spectra of methicillin-resistant and methicillin-susceptible strains grown in 20% D_2O for 4 hours with varied methicillin concentrations.

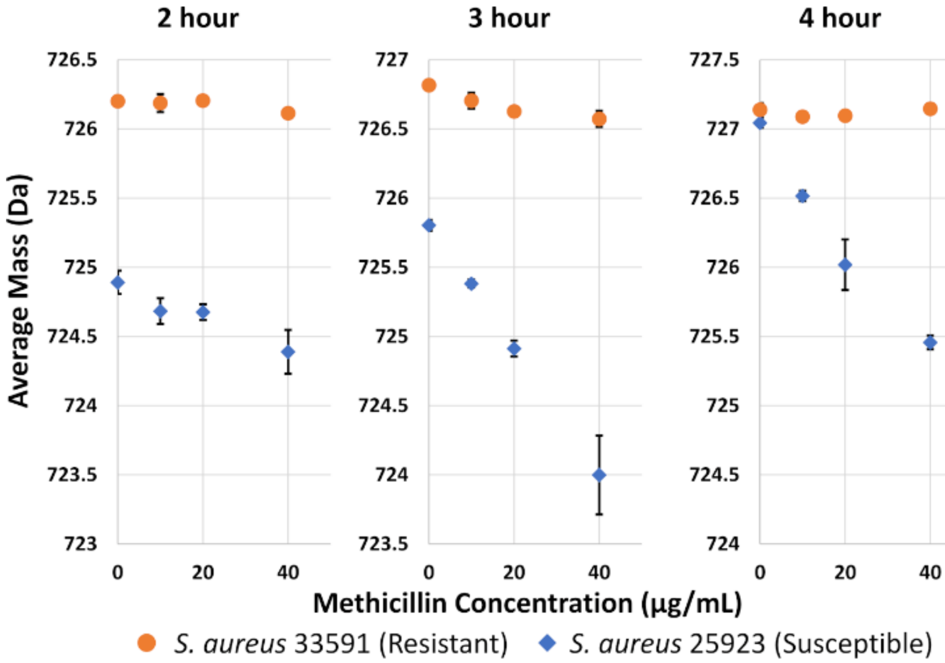

Figure S5. Average mass of PG 32:0 in *S. aureus* measured by the Orbitrap Q-Exactive HF after 20% D_2O microdroplet culture with varying methicillin concentrations and culture times.

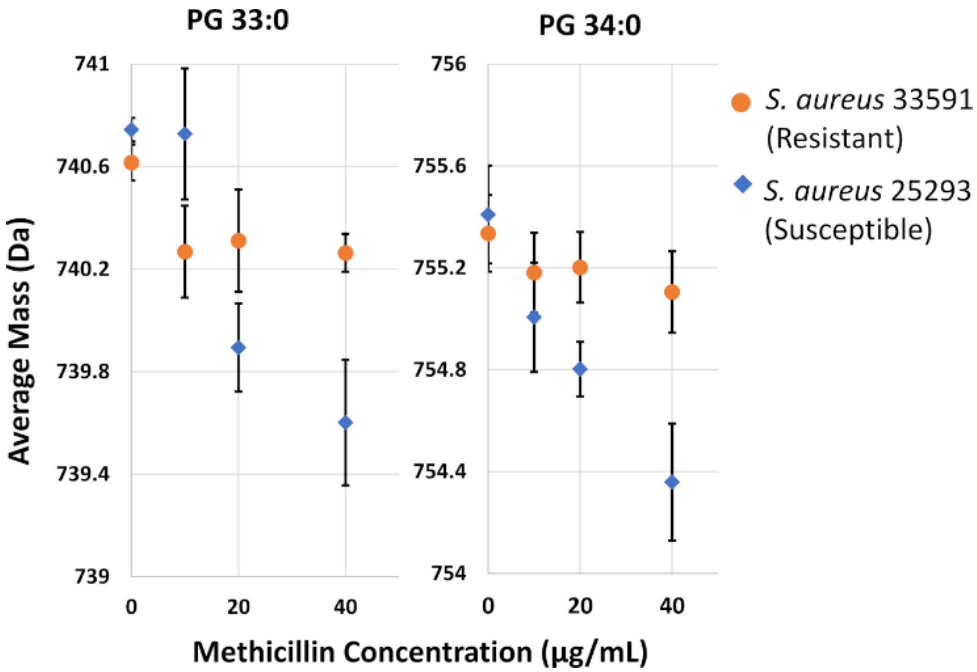

Figure S6. Average mass of low abundance PG 33:0 and PG 34:0 in *S. aureus* as measured by the Bruker Biotyper sirius after 20% D_2O microdroplet culture for 4 hours with varying methicillin concentrations.

Supplementary Table

Table S1. P-values for the average mass of PG 32:0 in *S. aureus* treated with varied methicillin concentration compared to untreated control. One-tailed T-test was used with unequal variance.

**Methicillin concentration**	**2 hour**		**3 hour**		**4 hour**	
	Susceptible	Resistant	Susceptible	Resistant	Susceptible	Resistant
10 µg/mL	0.181	0.192	0.0299	0.0802	0.0163	0.463
20 µg/mL	0.257	0.172	0.00235	0.111	0.00188	0.436
40 µg/mL	0.053	0.251	0.00183	0.081	0.000742	0.156
