# Supplementary material for: Rapid detection of antimicrobial resistance in methicillin-resistant Staphylococcus aureus using MALDI-TOF mass spectrometry
Source: Front Cell Infect Microbiol. 2023 Nov 23;13:1281155. doi: 10.3389/fcimb.2023.1281155 (PMC10702551; doi:10.3389/fcimb.2023.1281155)
Supplement: Supplementary file 2 [file DataSheet_2.zip › Data Labeling Legend.docx]

Data is labeled as strain_methicillin concentration (µg/mL)_replicate

M – MRSA strain

S – susceptible strain

Example

M_20_2 - MRSA strain, 20 µg/mL methicillin, replicate #2
